# Estimation of key indicators for bibliometric analysis in the applications of artificial intelligence in rheumatology

**DOI:** 10.1093/rap/rkaf079

**Published:** 2025-07-07

**Authors:** Maria Polyzou, Xenofon Baraliakos

**Affiliations:** Department of Pathophysiology, School of Medicine, National and Kapodistrian University of Athens, Laiko General Hospital, Athens, Greece; Rheumazentrum Ruhrgebiet, Ruhr-University Bochum, Herne, Germany; Rheumazentrum Ruhrgebiet, Ruhr-University Bochum, Herne, Germany

**Keywords:** artificial intelligence (AI), bibliometric analysis, Bradford’s Law, collaboration indicators, Lotka’s Law, rheumatology

## Abstract

**Objectives:**

Our aim was to estimate some interesting indicators regarding artificial intelligence (AI) applications in rheumatology literature published between 2010 and 2024 as well as to verify the application of Lotka’s law and Bradford’s law for the author’s scientific productivity in the field of these applications.

**Methods:**

A database was constructed using appropriate Scopus keywords related to the application of AI in the field of rheumatology and the indices were calculated using formulas found in relevant articles in the international literature. In addition, the applicability of Lotka’s law and Bradford’s law was used to evaluate the data of a bibliometric analysis in rheumatology.

**Results:**

The calculated indicators show the evolution and characteristics of publications in the scientific field under consideration. The results obtained show a high to moderate degree of author collaboration, while a small number of authors have published a relatively large number of articles. Also, a significant deviation was observed between the observed data and the ideal Lotka distribution, while the distribution of publications does not fit the Bradford distribution.

**Conclusion:**

The strong upward trend in the number of publications over the last 5 years indicates the great importance of AI in rheumatology. However, intensive work in this field is carried out by a few authors, who dominate scientific publications, which shows the reluctance of the majority of scientists to deal with the application of AI in rheumatology.

Key messages •  Artificial intelligence (AI) is becoming increasingly important in rheumatology research and clinical applications. •  A small number of authors have published a large share of the AI rheumatology research. •  Researchers in this field demonstrate a moderate to high level of collaboration.

## Introduction

The number of artificial intelligence (AI) applications in various fields of human life has been continuously increasing in the last decade and the applications of AI in the health sector are of great interest. Healthcare is rapidly evolving, driven by significant AI advancements, and rheumatology, a field characterized by diverse disorders, can benefit from these advancements [1]. AI is currently receiving a lot of attention, but there have been some objections to its feasibility and outcomes in medicine and other sciences. However, it is clear that AI can be used to improve the efficiency and effectiveness of healthcare and research [2].

Recently, AI has been integrated into the process of diagnosing and treating many diseases, including rheumatoid diseases [3, 4]. Many articles have been written in recent years on the application of AI in this sector, many of which concern bibliometric analyses [5]. Bibliometrics, a quantitative research technique for examining the academic qualities of literature, helps identify research hotspots and trends in a particular area and predict its future prospects [6]. Bibliometrics takes the external characteristics of scientific literature as research objects. More generally, bibliometrics and scientometrics are techniques that evaluate published research both qualitatively and qualitatively [7]. One of the main areas in bibliometrics research concerns the estimations of useful indices as well as some bibliometrics laws.

This article deals with estimating indicators concerning applications of AI in the rheumatology literature published from 2010 to 2024. These indicators are numerical data that represent different characteristics of scientific activity related to both the production and use of information. Another purpose of the article is to verify the applicability of Lotka’s law and Bradford’s law of scattering regarding applications of AI in rheumatology.

There have been many studies conducted in the last 3 years investigating the general bibliometric characteristics of publications related to the use of AI in rheumatology. The most characteristic is the study conducted by Zhang *et al.* [6], which presents the results of a bibliometric analysis on the applications of AI in RA. Specifically, this study analysed the characteristics of publications related to AI research in RA, the overall distribution of annual publications in this scientific field, the countries with the most publications, the top institutions and authors, active journals, relevant citations and keywords. As mentioned above, the present study mainly focuses on calculating some basic indicators regarding the applications of AI in rheumatology, which are different from the indicators of other related bibliometric surveys.

## Description of key indicators

### Collaboration index (CI)

The CI is the simplest index presently used to explore the literature, and is interpreted as the mean number of authors per paper. It is calculated by a mathematical formula that was suggested by Lawani [8]. Supposing collection A of the research papers published in a discipline or in a journal during a certain period of interest, then the CI is calculated by the following formula:


(1) $$CI=\frac{\sum_{j=1}^{A}jf_{j}}{N},$$

where

*f_j_* = number of *j* authored papers published in the discipline during a certain period of time,

*N* = total number of research papers published in the discipline during a certain period of time,  $N=\sum_{j=1}^{A}f_{j}$ and

*A* = total number of authors in the collection.

The key features of CI are it is a measure of the average number of authors, is easily calculated, but not easily interpreted as a degree, and it gives zero weight to single-authored articles, which refer to no collaboration [9].

### Degree of collaboration (DC)

The DC is defined as the ratio of the number of collaborative research papers to the total number of research papers in the discipline during a certain period of time [10]. The formula is expressed as:


(2) $$DC=\frac{N_{m}}{N_{m}+N_{s}},$$

where

*N*_m_ = number of multi-authored papers in the discipline published during a year and

*N*_s_ = number of single-authored papers in the discipline published during a year.

### Collaboration coefficient (CC)

The CC was suggested to remove the weaknesses of the DC to differentiate among levels of multiple authorships. The CC is used to measure the extent and strength of collaboration among the selected rheumatologic journals. It can be expressed mathematically as follows [11]:


(3) $$CC=1-\frac{\sum_{j=1}^{A}(\frac{1}{j})f_{j}}{N},$$

where

*j* = number of the authors in a paper, i.e. 1, 2, 3, …,

*f_j_* = number of *j* authored research papers,

*N* = total number of *j* research papers published in a year and

*A* = total number of authors per paper.

### Lotka’s law

Lotka’s law is a foundational principle in bibliometrics that describes the relationship between the frequency of scientific output (publications) and the number of contributing authors in a given field [7, 12]. It is known as the ‘inverse square law of scientific productivity’ and is a principle in bibliometrics that describes the distribution of scientific productivity among authors. The original statement of Lotka’s law is as follows: the number of authors who have published *x* papers occupied the proportion of total authors within a certain period; the proportion is denoted as f(*x*), which varies inversely as the square. In other words, the number of authors producing *x* publications is inversely proportional to *x*^2^, meaning that a small number of authors produce a disproportionately high number of publications, while a majority contribute only a few [13]. The Lotka’s law is mathematically expressed by the general relationship


(4) $$y=\frac{c}{x^{n}},$$

where

y = number of the authors contributing *x* publications,

*c* = a constant and

*n* = a parameter that is typically expected to be close to 2 for an ideal Lotka’s law distribution.

The significance of this law lies in its ability to model the concentration of scientific contributions, providing insights into the distribution of productivity across different authors and allowing researchers to identify leading contributors in a domain. Estimation of parameter *n* is the first step in the application of Lotka’s law. The value of *n* can be estimated by using the linear least squares (LLS) regression method or one of its equivalent forms given by the following equation:


(5) $$n=\frac{N*\Sigma X\Upsilon -\Sigma X*\Sigma \Upsilon }{Ν*{\Sigma X}^{2}-{\left(\Sigma X\right)}^{2}},$$

where

*N* = number of pairs of data considered, *x* = 1, 2, 3, …, *x*_max_,

*X* = logarithm of *x*, i.e. number of articles, and

*Y* = logarithm of *y*, i.e. number of authors.

In this article, the parameter *n* was calculated using equation (5). This equation allows one to estimate the slope of the observed distribution based on the log-transformed values of *x* and *y*, included in Supplementary Table S1, available at *Rheumatology Advances in Practice* online. The logarithmic transformations serve to linearize the power law relationship, making it easier to perform regression analysis.

### Bradford’s law

Bradford’s law of scattering describes a quantitative relation between articles belonging to the same scientific field published in different journals. It is a rule of thumb in scientific journal bibliographies, which mainly reflects the unbalanced distribution of articles. Bradford’s law ranks scientific journals in descending order by the productivity of articles on a particular topic and identifies a core of journals that were most devoted to that topic [12].

According to this law, if the journals are divided into groups, each containing the same number of articles on a given subject, then the number of journals in the succeeding groups forms a geometric progression [14]. Bradford did not provide a mathematical model, but models were proposed by Brooks, Vickery and Leimkuhler [15]. The Leimkuhler model is then explained using the Bradford law formulation vocabulary. Leimkuhler explained how Bradford distribution operates as follows:

If *R*(*r*) refers to the sum of articles produced by the journal of rank 1, 2, 3, …, *r* then


(6) R(r)=alog(1+br),

where a, b are constants.

Later, Egghe [3] explained the Leimkuhler model demonstrating that its constants (a and b) may be modelled as follows:


(7) $$a=\frac{Y_{0}}{\;\text{log}\;k}$$

(8) $$b=\frac{k-1}{r_{0}},$$

where *r*_0_ is the number of sources in the first Bradford zone, *Y*_0_ is the number of items in each Bradford zone and *k* is Bradford multiplier.

The Bradford multiplier is calculated as [14]:


(9) $$k={\left(e^{g}Y_{m}\right)}^{\frac{1}{p\prime }},$$

where *Y*_m_ is the number of items in the most productive source, *g* is the Euler number and *e^g^* = 1.781.

The previous equation can be rewritten as follows:


(10) $$k={\left(1.781Y_{m}\right)}^{\frac{1}{p\prime }}$$

and *Y*_0_ is calculated as:


(11) $$Y_{0}=\frac{A}{p},$$

where *A* is the total number of articles.

Let *T* be the total number of journals, and we have *p* groups, then *T* can be expressed as:


(12) $$T=r_{0}+r_{0}k+r_{1}k^{2}+\cdots +r_{0}k^{p-1}$$

or


(13) $$r_{0}=\frac{T(k-1)}{k^{p}-1},$$

thus


(14) $$r_{0}=\frac{T}{1+k+k^{2}+\cdots +k^{p-1}}.$$

Since *A* and *T* are both known from the database, it is easy to calculate *y*_0_ and *r*_0_ using the previous equations.

## Data, estimations and results

### Dataset

In this article, the methodology and data analysis were executed using the VOSviewer tool (https://www.vosviewer.com/) and the Python programming language (https://www.python.org/). The survey covers the time frame from 1 January 2010 to 31 December 2024. The construction of the database was based on the use of Scopus keywords, and detailed information about the database is presented in Supplementary Table S1, available at *Rheumatology Advances in Practice* online. Using the Scopus database, an extensive search was conducted for English-language articles focusing on applications of AI in rheumatology. Non-English articles were excluded using filters in the Scopus database.

The Scopus database was searched for articles that include at least one term from the group ‘artificial intelligence’ and ‘machine learning’ and at least one term from the group ‘rheumatology’, ‘rheumatoid’, ‘arthritis’, ‘osteoarthritis’, ‘spondyloarthritis’ and ‘rheumatic’, along with the word ‘diseases’. This means that an article was included only if it mentioned both a concept from AI and a concept from rheumatology.

**Figure 1.**
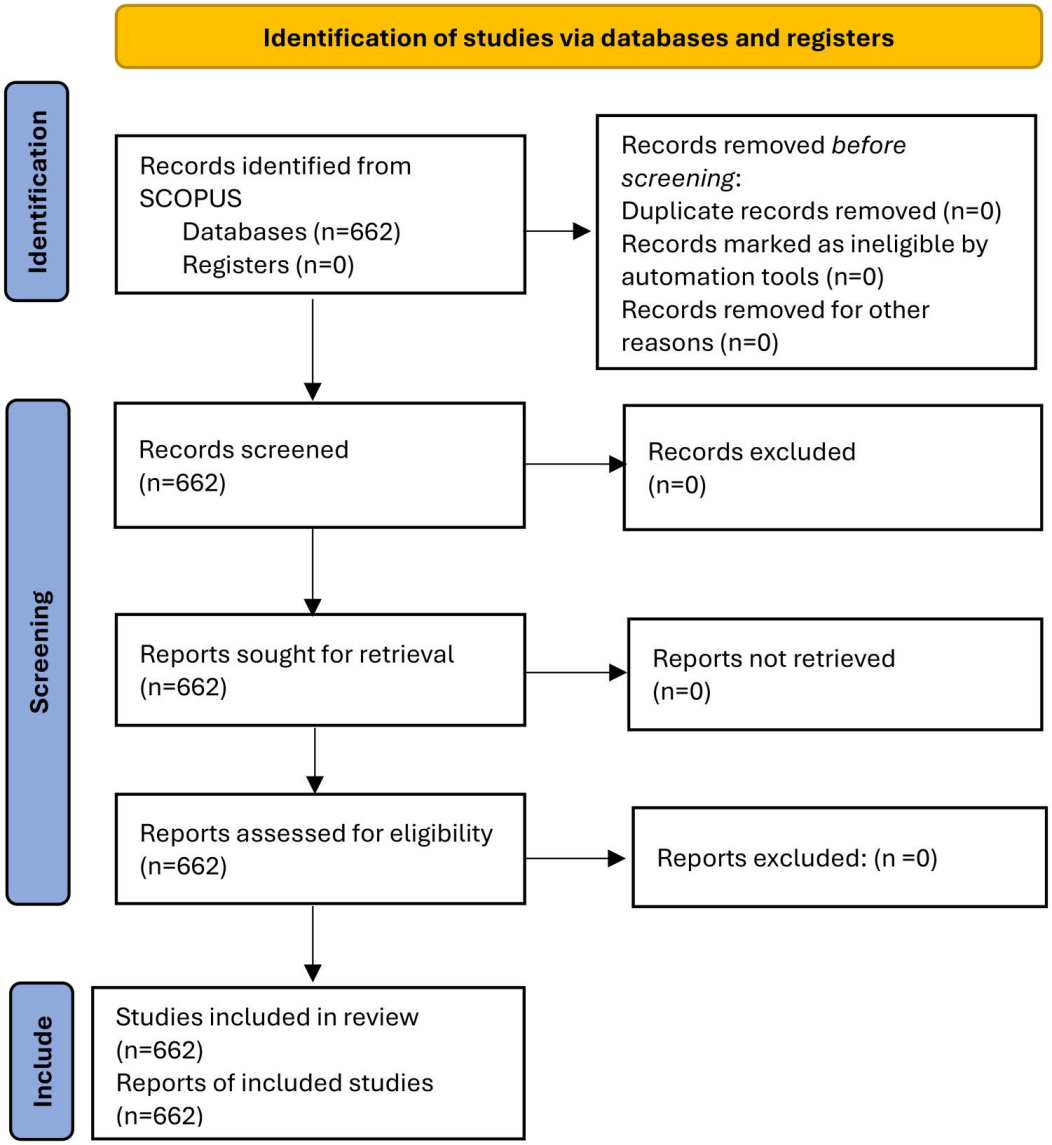
Flow chart of the study selection process

The study selection process is illustrated in the flow chart (Fig. 1), which was created according to the Preferred Reporting Items for Systematic Reviews and Meta-Analyses (PRISMA) guidelines [16]. It is important to note that, in contrast to typical systematic reviews, this bibliometric analysis did not involve a stage-by-stage assessment of the full content of individual articles for inclusion. Instead, the depicted flow reflects the data retrieval process from the Scopus database, where initial records (*n* = 662) were identified using a predefined query and subsequently refined through the application of database filters (as detailed in Supplementary Table S1, available at *Rheumatology Advances in Practice* online). While the PRISMA flow chart provides a structured overview of the data retrieval, its direct applicability is limited by the inherent constraints of bibliometric analyses, which focus on metadata rather than in-depth content evaluation.

Ethical considerations were adhered to throughout the study process, ensuring respect for copyright and citation norms while utilizing data from existing literature. After the initial search, the extracted data were exported to a text file for further analysis.

The decision to start the analysis in 2010 was motivated by the objective to capture a thorough and contemporary understanding of the subject. This starting point was chosen because advances in technology affecting medicine have been particularly intense in recent years [6].

**Figure 2.**
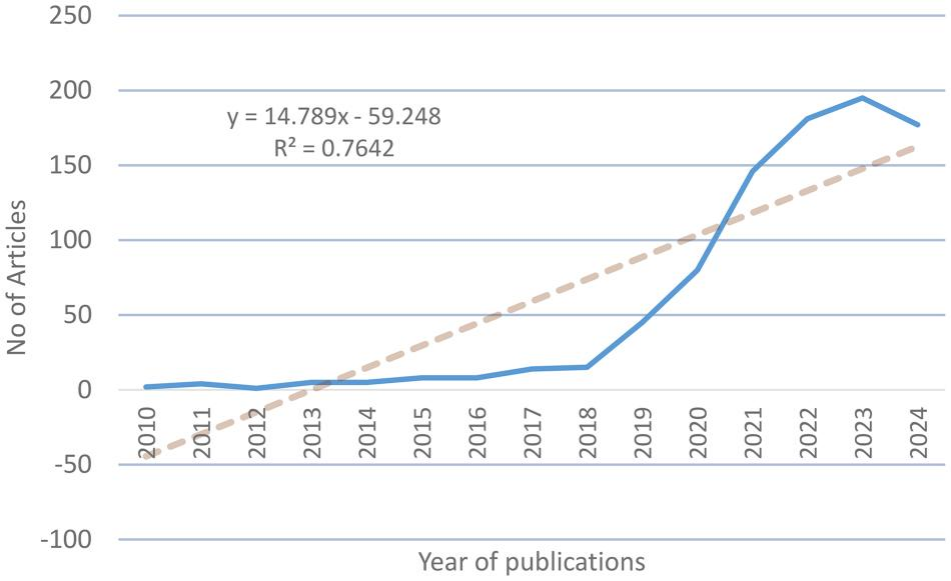
The evolution of the number of publications concerning the use of AI in rheumatology in the period 2010–2024

Fig. 2 presents the evolution of the publications that appeared during the research period and are related to the scientific field under consideration. As illustrated in this diagram, there was a significant increase in publications after 2018, which shows the particular use of AI in rheumatology. The small decrease that appears in 2024 is due to the fact that the publication data used does not include some journals that had not been published by the date of the search. Similar is the evolution of the number of publications found during the period 2003–2022 in the article by Zhang *et al.* [6].

### The CI

**Figure 3.**
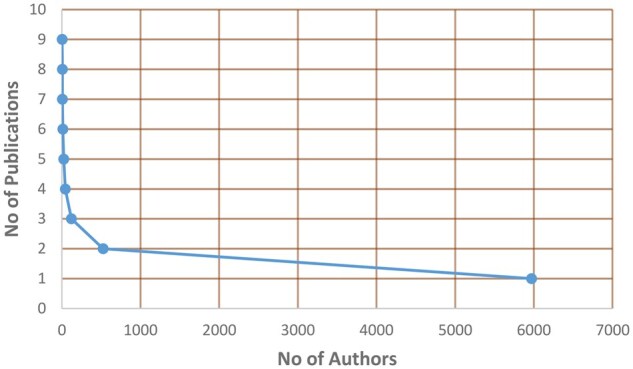
The relationship between the number of the authors and the number of their publications concerning the use of AI in rheumatology in the period 2010–2024

Supplementary Table S2 (available at *Rheumatology Advances in Practice* online) presents the number of publications and the corresponding number of authors in the field of AI applications in rheumatology for the aforementioned time period. In addition, Supplementary Table S3, available at *Rheumatology Advances in Practice* online, contains the number of co-authors *j* and the corresponding number of articles *f_j_*. Using the data in Supplementary Table S2, available at *Rheumatology Advances in Practice* online, the diagram in Fig. 3 was constructed, which shows the relationship between the number of authors and the number of publications that each author has in the scientific field under consideration. The conclusion that emerges from the data in Supplementary Table S2, available at *Rheumatology Advances in Practice* online, and Fig. 3 is that a small number of authors have more than three publications. This can be justified by the fact that the use of AI in rheumatology and the healthcare sector in general is intense after 2019, which is evident in Fig. 1.

**Figure 4.**
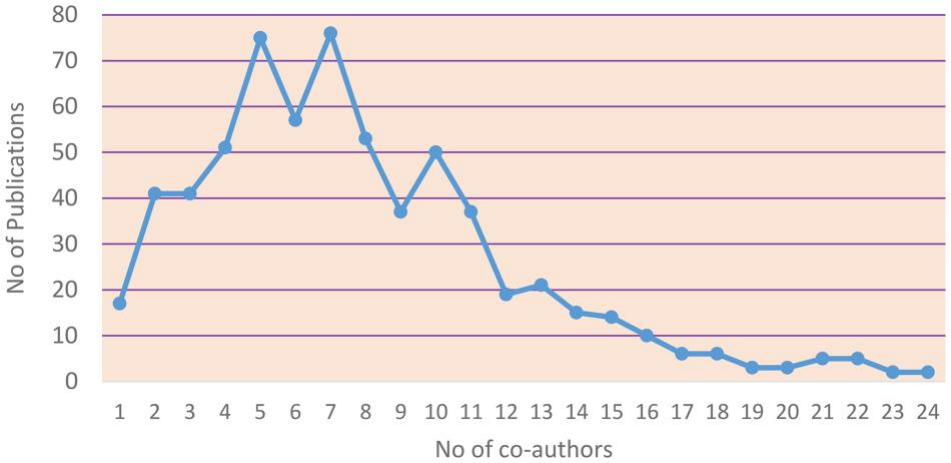
Number of co-authors and number of corresponding publications

Applying equation (1), we calculated the CI corresponding to the data in Supplementary Table S2, available at *Rheumatology Advances in Practice* online, as 8.02. We estimate that the CI value is high, which indicates the high degree of collaboration of scientists in the researched scientific field. In addition, for a better overview using the data in Supplementary Table S3, available at *Rheumatology Advances in Practice* online, Fig. 4 is presented, which also depicts the degree of collaboration between authors. As seen in Fig. 4, in most articles from 3 to 10 authors collaborate.

### The DC

Using equation (2) and the data of Supplementary Table S3, available at *Rheumatology Advances in Practice* online, the DC is calculated. In equation (2), *N*_m_ = 645 and *N*_s_ = 17 and therefore DC = $\frac{645}{645+17}=0.974.$ 

### The CC

The CC is a measure of collaboration in research that reproduces in the mean number of authors per publication and the proportion of multi-authored papers. By using equation (3) and data derived from appropriate processing of the data in Supplementary Table S3, available at *Rheumatology Advances in Practice* online, we calculated the coefficient of cooperation, which is equal to CC = 0.94.

The values of the estimated coefficients lead to the conclusion that the degree of collaboration of authors in writing articles in the scientific field under consideration is high. In general, collaborative research seems to be increasing in popularity, and this may be due to several factors. In many countries it is actively supported by governments and organizations. However, the decision to conduct research with partners instead of individually influences the research process as well as its results [17]. Furthermore, the interdisciplinary nature of the field explored in this article favours collaboration between scientists, who belong to the health, informatics and statistical disciplines.

### Implementation of Lotka’s law

The data presented in Supplementary Table S4, available at *Rheumatology Advances in Practice* online, provide an overview of the distribution of scientific productivity, with *x* representing the number of publications and *y* indicating the number of authors with *x* publications. These values are derived from a bibliometric analysis performed using VOSviewer.

**Figure 5.**
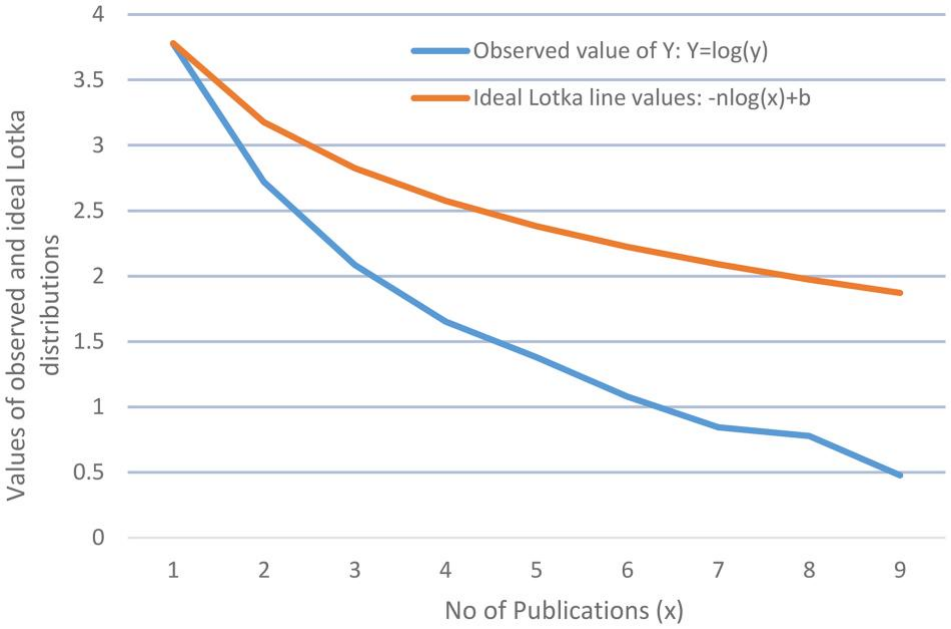
Comparison of the values of observed and ideal Lotka’s law distribution—log–log plot of the number of publications *vs* the number of authors concerning the use of AI in rheumatology in the period 2010–2024

The total value of *n* for the present data, as shown in Supplementary Table S1, available at *Rheumatology Advances in Practice* online, was calculated using equation (5) as −3.40. This value diverges significantly from the expected ideal value of −2 under Lotka’s law, suggesting that the dataset does not conform to the typical Lotka distribution. To further investigate this deviation, Fig. 5 was constructed, which presents a log–log plot comparing the observed and ideal Lotka distributions. The observed regression line, which is derived from linear regression on the log-transformed data, is juxtaposed with the ideal Lotka line. The latter is modelled with a theoretical slope of −2, representing the expected distribution according to Lotka’s law. The observed regression line was calculated using the next equation, which is derived from equation (4):


(15) log(y)=−nlog(x)+b,

where

*n* = the slope derived from the dataset, which is −3.40, and

*b* = the intercept, calculated as 3.78 for this dataset.

The diagram clearly demonstrates a significant deviation between the observed data and the ideal Lotka distribution. While the ideal Lotka line exhibits a steeper slope of −2, indicating a more equitable distribution of publications among authors, the observed data reveal a slope of −3.40. This slope indicates that there is a higher-than-expected concentration of publications among prolific authors, with fewer authors contributing minimally compared with what Lotka’s law predicts.

In general, a grouping of the results obtained from applying Lotka’s law to a dataset of publications and their corresponding authors leads to three main categories: a large number of authors with one or very few publications, a rapid decrease in authors with a concomitant decrease in the number of their publications and a small number of authors who have authored a large number of publications, constituting a ‘core of highly productive authors’.

The negative value of *n* further supports this finding, indicating that the distribution of author productivity in this dataset is more skewed than expected. Such deviations from Lotka’s law can occur due to several factors, including increased barriers to publication, an environment that disproportionately favours highly productive authors or unique characteristics of the field under study. The data suggest that a small group of highly prolific authors dominate the scientific output while the majority of authors contribute only a few publications, a pattern that deviates from the standard Lotka distribution.

Thus, according to the previous analysis and the results found, we found that the relationship between the number of authors and publications in the field under investigation corresponds to the third of the above categories. The interpretation that can be given for this deviation from Lotka’s law is related, on the one hand, to the short period, just the last 5 years, during which the majority of the articles appeared and, on the other hand, to the particular characteristics of the scientific field. As mentioned above, the emergence of AI techniques is recent and, as with any innovation, in the case under investigation there is an initial reluctance of scientists to apply these techniques [18, 19]. Furthermore, we hypothesize that the reluctance of physicians is greater than that of scientists in other fields, due to their lower daily involvement with the subject of computing. Despite the fact that the integration of informatics into healthcare is now considered essential, a large number of physicians are not comfortable with its use, still relying on traditional clinical practices.

### Implementation of Bradford’s law

Focusing on assessing the verbal formulation of Bradford’s law, Supplementary Table S5, available at *Rheumatology Advances in Practice* online, was constructed in which a comprehensive summary of journal documents is presented. The number of journals is arranged by a decreasing number of documents. To test the verbal formulation of Bradford’s law, the rank number of journals, number of documents and cumulative documents are given.

For testing the algebraic interpretation of the law, the 160 journals were divided into three zones. The Bradford’s multiplier factor was arrived at by dividing the journals of a zone by its preceding zone. Supplementary Table S5, available at *Rheumatology Advances in Practice* online, serves as a valuable reference to evaluate the applicability of Bradford’s law and analyse document patterns literature that belongs in the field of AI and rheumatology.

In the present dataset, 12 journals account for 225 articles, the next 37 journals account for 217 articles and the next 111 journals account for 220 articles. In other words, one-third of the total articles have been covered by each group of journals. According to Bradford’s law, the zones identified in this way form an approximately geometric sequence of the form 1:*n*:*n*^2^ [20–22]. However, the ratio of each band in the present study was found to be 12:37:111, a ratio that does not fit the Bradford distribution. Therefore, the following method based on the Leimkuhler model was used to verify the Bradford scattering law.

Based on what was mentioned above and the data that were executed using the VOSviewer tool as well the Python programming language, we have the following variables: *A* = 662 (total number of articles), *T* = 160 (total number of journals), *p* = 3 (groups), *Y*_m_ = 51 (the number of items in the most productive source) and *e^g^* = 1.78. Using these values and the above equations, we find the following:


$$\begin{array}{c} k={\left(1.781*51\right)}^{\frac{1}{3}}=4.42\\ r_{0}=\frac{160(4.42-1)}{{\left(4.42\right)}^{3}-1}=6.41\\ Y_{0}=\frac{662}{3}=220.66\\ a=\frac{220.66}{\;\text{log}\;4.42}=341.84\\ b=\frac{4.42-1}{6.41}=0.533 \end{array}$$

Using the above values, we estimated the distribution:


$$\left(r_{0}\right):\left(r_{0}\times k\right):\left(r_{0}\times k^{2}\right)=\left(6.41\right):\left(6.41\times 4.42\right):\left(6.41\times 4.42^{2}\right)=\left(6.41\right):\left(28.33\right):\left(125.22\right).$$

The findings of the calculations are shown in Supplementary Table S6, available at *Rheumatology Advances in Practice* online, and it is clear that the journals contributing articles to each zone increase by a multiplier of 4.42. Top was 6.41, with ≈6 journals appearing in the nucleus zone contributing 164 articles, followed by 28.33, with ≈28 journals in the second zone containing 189 articles, and 125.22, with ≈125 journals containing 309 articles in the third zone. Since the percentage of error is negligible, Bradford’s law fits well in this dataset.

**Figure 6.**
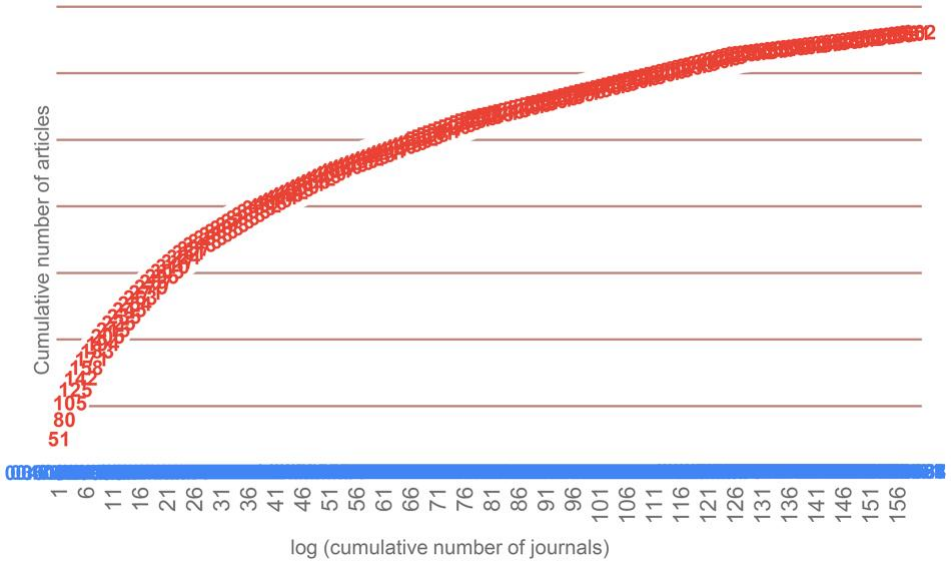
Bradford graphs of article distribution in journals concerning the use of AI in rheumatology in the period 2010–2024

Figure 6 was then constructed, showing the logarithmic plot of the cumulative number of journals on the horizontal (*x*) axis and the cumulative number of articles on the vertical (*y*) axis. If the distribution confirms Bradford’s law, it will show three characters [20, 21]: (i) a rapid increase at the beginning, shows the core journals, (ii) a linear growth in the middle indicating strong relation between the variables and (iii) a decrease at the endpoint that denotes incompleteness of the bibliography verified.

It can be observed from the diagram that fewer journals have a very high level of concentration in productivity. The journals that show the most articles in the scientific field under investigation are Frontiers in Immunology, Arthritis Research and Therapy, Scientific Reports, PLoS One, RMD Open and Osteoarthritis and Cartilage. These journals constitute 3.7% of all journals and contain 23% of the articles.

## Conclusions

In this article, some key indicators regarding the applications of AI in the rheumatology literature published from 2010 to 2024 were calculated. These indicators show the evolution as well as the characteristics of publications in this scientific field. From the preceding analysis and the calculations made, it follows that the degree of collaboration of the authors can be characterized as relatively high. Furthermore, the relationship between the authors and the number of their publications analysed shows that a small number of authors have published a relatively large number of articles.

Regarding the number of publications in the scientific field under consideration, it has been increasing in the last 5 years, which shows the great importance of AI in rheumatology. Also, the data used show a significant deviation between the observed data and the ideal Lotka distribution. A small group of very productive authors dominates the scientific output, with the majority of authors contributing only a few publications, a pattern that deviates from the typical Lotka distribution. Finally, based on the survey data, the distribution of publications does not fit the Bradford distribution, while application of the Leimkuhler model showed a better fit.

In summary, the general conclusions arising from the quantitative analysis that preceded are the following:

- The use of AI in rheumatology has shown increasing trends in the last 5 years according to the number of relevant articles published in scientific journals during the period 2010–2024. This trend is considered logical and expected, given that developments in information technology and applications of AI have been rapid in this 5-year period.
- The degree of collaboration of the authors in the above articles, according to the calculated indicators, is relatively high. As mentioned above, the scientific subject of AI and its applications is characterized by an interdisciplinary nature that requires the collaboration of scientists with different backgrounds.
- The clear deviations from Lotka’s law and the more general results from the preceding analysis led to the conclusion that a small number of authors show high productivity. In contrast, there is a large number of authors with few publications.
- The findings of the calculations resulting from the application of Bradford’s law showed that a small number of journals concentrate the largest percentage of articles. The interpretation of this result is related to the specificity of the relationship between AI and the health sector. As mentioned above, a small number of authors have intensively dealt with the subject, which has resulted in their concentration in a small number of journals.

## Supplementary Material

rkaf079_Supplementary_Data

## Data Availability

The data underlying this article were obtained using keywords from the Scopus database. Data analysis was performed using the VOSviewer tool and the Python programming language. An extensive search was conducted for English-language articles focusing on applications of AI in rheumatology. Non-English-language articles were excluded using filters in the Scopus database. Aggregate results supporting the findings are available from the corresponding author upon reasonable request.
