# Supplementary material for: Estimation of key indicators for bibliometric analysis in the applications of artificial intelligence in rheumatology
Source: Rheumatol Adv Pract. 2025 Jul 7;9(3):rkaf079. doi: 10.1093/rap/rkaf079 (PMC12321292; doi:10.1093/rap/rkaf079)
Supplement: rkaf079_Supplementary_Data [file rkaf079_supplementary_data.docx]

**Supplementary material**

**Supplementary Table S1: Data retrieval constraints and parameters for the Scopus database**

| **Data** | **Search criteria and results** |
| --- | --- |
| Database / Search field | Scopus / Title, Abstract, Keywords |
| Keywords | Artificial Intelligence, machine learning, Rheumatology, rheumatoid arthritis, Osteoarthritis, spondyloarthritis, rheumatic diseases |
| Author name - Subject Area - Source title – Affiliation - Funding sponsor - Country | All |
| Document type/ Source type | Article / Journal |
| Language / Publication stage | English / Final |
| Open Access | All open access |
| Scopus query | TITLE-ABS-KEY ( "artificial intelligence" , OR "machine learning" , AND "rheumatology" , OR "rheumatoid" , OR "arthritis" , OR "osteoarthritis" , OR "spondyloarthritis" , OR "rheumatic" , AND "diseases" ) AND PUBYEAR > 2009 AND PUBYEAR < 2025 AND ( LIMIT-TO ( DOCTYPE , "ar" ) ) AND ( LIMIT-TO ( SRCTYPE , "j" ) ) AND ( LIMIT-TO ( LANGUAGE , "English" ) ) AND ( LIMIT-TO ( PUBSTAGE , "final" ) ) AND ( LIMIT-TO ( OA , "all" ) ) |
| Data extraction period | January 1 2010- December 31 2024 |
| Number of publications | 662 |

**Supplementary Table S2: Authors and the number of their publications**

| Authors | 5972 | 526 | 122 | 45 | 24 | 12 | 7 | 6 | 3 |
| --- | --- | --- | --- | --- | --- | --- | --- | --- | --- |
| Number of publications | 1 | 2 | 3 | 4 | 5 | 6 | 7 | 8 | 9 |

**Supplementary Table S3: Number of co-authors and number of corresponding publications**

| Authors j | 1 | 2 | 3 | 4 | 5 | 6 | 7 | 8 | 9 | 10 | 11 | 12 | 13 | 14 | 15 |
| --- | --- | --- | --- | --- | --- | --- | --- | --- | --- | --- | --- | --- | --- | --- | --- |
| Publications fj | 17 | 41 | 41 | 51 | 75 | 57 | 76 | 53 | 37 | 50 | 37 | 19 | 21 | 15 | 14 |
| Authors j | 16 | 17 | 18 | 19 | 20 | 21 | 22 | 23 | 24 | 25 | 26 | 27 | 28 | 29 | >29 |
| Publications fj | 10 | 6 | 6 | 3 | 3 | 5 | 5 | 2 | 2 | 2 | 3 | 1 | 1 | 1 | 8 |

**Supplementary Table S4: Distribution of Authors by Number of Publications and Corresponding**

| Publications (x) | Authors with x publications  (y) | The logarithm value of x (X) | The logarithm value of y (Y) | XY | X^2^ | n |
| --- | --- | --- | --- | --- | --- | --- |
| 1 | 5972 | 0,00 | 3,78 | 0,00 | 0,00 |  |
| 2 | 526 | 0,30 | 2,72 | 0,82 | 0,09 |  |
| 3 | 122 | 0,48 | 2,09 | 1,00 | 0,23 |  |
| 4 | 45 | 0,60 | 1,65 | 1,00 | 0,36 |  |
| 5 | 24 | 0,70 | 1,38 | 0,96 | 0,49 |  |
| 6 | 12 | 0,78 | 1,08 | 0,84 | 0,61 |  |
| 7 | 7 | 0,85 | 0,85 | 0,71 | 0,71 |  |
| 8 | 6 | 0,90 | 0,78 | 0,70 | 0,82 |  |
| 9 | 3 | 0,95 | 0,48 | 0,46 | 0,91 |  |
| **Total** | **6717** | **5,56** | **14,80** | **6,49** | **4,22** | **-3.40** |

**Supplementary Table S5: Distribution of authors by number of articles**

| **Zones** | **No of journals** | **No of articles** | **% of journals** | **% of articles** | **Bradford Multiplier** |
| --- | --- | --- | --- | --- | --- |
| 1 | 12 | 225 | 7.5 | 33.60 | - |
| 2 | 37 | 217 | 23.1 | 33.10 | 3.08 |
| 3 | 111 | 220 | 69.34 | 33.30 | 3.00 |
| **Total** | **160** | **662** | **100** | **100** | **3.04 (mean)** |

**Supplementary Table S6: Scattering of Journals and Articles over Bradford zone**

| **Zones** | **No of journals** | **No of articles** | **% of journals** | **% of articles** | **Bradford Multiplier** |
| --- | --- | --- | --- | --- | --- |
| 1 | 6.41 | 164 | 4.00 | 24,78 | - |
| 2 | 28.33 | 189 | 17.7 | 28,55 | 4.42 |
| 3 | 125.22 | 309 | 78.3 | 46,87 | 4.42 |
| **Total** | **160** | **662** | **100** | **100** | **-** |
